# Using Epidemiological Test Diagnostics to Select Fraud Detection Methods: Secondary Analysis of Quantitative Cross-Sectional Survey Data

**DOI:** 10.2196/85161

**Published:** 2026-03-05

**Authors:** Rachel Willard-Grace, Tali Klima, Mansi Dedhia, Emily Lo, Annie Nisnevich, Allison Gray, Holly Henry

**Affiliations:** 1 Department of Family and Community Medicine, University of California, San Francisco, Box 1315 2540 23rd St., Floor 5, 5511, San Francisco, CA, 94143, United States, 1 415-297-3969; 2 Practical Research Solutions, Sunnyvale, CA, United States; 3 Lucile Packard Foundation for Children's Health, Palo Alto, CA, United States

**Keywords:** health care surveys, bots, surveys, questionnaires, survey methodology, web-based data collection, specificity, sensitivity

## Abstract

**Background:**

Survey research has the potential to elevate the experiences and opinions of marginalized populations. The rising number of bot attacks, a method of participant fraud that creates multiple records in survey data using automated software, threatens to drown out those voices and produce inaccurate findings. Rapid identification and mitigation of bot attacks are vital; however, there is limited guidance for researchers on scalable approaches to address this problem.

**Objective:**

This study aimed to assess how well recommended methods detect fraud using an epidemiological diagnostic test framework to inform web-based survey researchers on how best to identify and shut down bot attacks.

**Methods:**

We analyzed data from a cross-sectional web-based statewide survey on access to pediatric subspecialty care in California that used Qualtrics survey software. Caregivers of children with chronic conditions were recruited through family resource centers (FRCs), nonprofit agencies serving families with developmental delays and chronic medical conditions. The survey was sent out to 17 FRCs, whose staff distributed anonymous links to their clients through listservs and flyers. Respondents who completed the survey received a US $30 gift card. Prior to launch, we designed a protocol to identify and respond to bot attacks and reviewed responses for markers of fraudulent activity. If markers were identified or there was a spike in responses, a senior member of our research team reviewed patterns among all submitted surveys for each FRC to look for signs of bot attacks. We calculated epidemiologic measures of diagnostic test accuracy, such as sensitivity, specificity, positive predictive value, and negative predictive value, which describe a test’s ability to distinguish “disease” (in this case, fraudulent records) from normal cases, to better understand the utility of recommended strategies to identify bot attacks.

**Results:**

We received 646 valid survey records and 905 fraudulent records resulting from bot attacks. The primary indicator of a bot attack was a sudden spike in responses to the survey. Differences in demographics and outcomes, including wait times for pediatric subspecialty care and use of health care services, between the valid and fraudulent data indicated that failure to remove fraudulent records would have substantially altered the survey results. Most recommended methods in the literature for identifying fraudulent responses had low sensitivity to detect bot attacks, and only 2 were better than chance alone at correctly identifying bot attacks. Combinations of fraud markers and blocks of repeated responses were particularly useful to identify bot attacks.

**Conclusions:**

Fraudulent data entry using bots is increasing in survey research. Sharing flexible protocols to identify and mitigate them in a way that is responsive to their ever-changing nature is vital to ensuring that researchers elevate the voices of real people within survey research to inform policy and programmatic discussions.

## Introduction

Survey research has the potential to elevate the experiences and opinions of individuals who may be difficult to reach. While ideal survey methods include the use of individualized, single-use survey links shared with a defined and verified set of respondents, in reality, there are important populations who are not neatly vetted and included in registries and, in some cases, whose safety depends on being invisible and unregistered [1,2]. Since creating single-use survey links matched to known populations is challenging in these situations, many surveys use generic survey links, which carry a greater risk of use by fraudulent responders for profit or manipulative purposes.

The rising number of fraudulent responses threatens to drown out those voices. Our nonexhaustive literature review of 13 studies exploring fraudulent responses found a range of 13% to 97% of survey responses were identified as fraudulent, with a pooled proportion of 61%. Eight of 13 studies reported that more than half of responses were fraudulent (Multimedia Appendix 1) [1-10]. The onslaught of fraudulent responses has a number of consequences; key among these is the distortion of study findings, leading to inaccurate results if fraudulent records are not excluded [4]. For the purpose of this paper, we define a “fraudulent record” as any record in which false information is presumed to be purposely provided. Bot attacks comprise a subset of these fraudulent records and are distinguished by the use of software to generate a large number of responses [11].

Rapid identification and mitigation of bot attacks are vital; however, there is limited guidance for survey researchers in the face of rapidly evolving and ever-more-sophisticated fraudulent attacks. We did not find any prior studies that used standard epidemiological methods to assess the effectiveness of various bot detection approaches. Standard recommendations include identifying duplicate responses or off-topic responses, inconsistencies between location stamp and location reported, similar or identical response or pattern of responses is provided across surveys, identifying inconsistencies between different survey items (eg, age and year of birth), embedding “honeypot” items that are visible to bots but not humans, or short survey completion time [10]. Most of these recommendations are based on assumptions of manual fraud, where individuals submit multiple surveys by hand (formerly known as “ballot box stuffing”), or relatively unintelligent technology that uses simple strategies and does not learn how to game the survey (eg, overcome screening items).

Based on our experience and anecdotal evidence from others, researchers have already observed failures of many of these methods, which are less applicable to modern bot capabilities. Moreover, among survey researchers seeking to reach populations with limited digital literacy, some suggested methods pose the additional danger of excluding eligible but less digitally savvy participants [6]. For example, we abandoned use of CAPTCHA screening (identification of images, such as motorcycles, in a matrix) on our survey platform after technologically savvy staff were consistently unable to pass the screening themselves.

Little is known about the ability of current fraud detection methods to successfully identify fraudulent records (sensitivity, in clinical epidemiological terms) or their ability to correctly identify valid records (ie, not to label a valid record as a fraudulent one, hereafter referred to as “specificity”). Using data from a California-wide survey and a diagnostic approach that treats bot attacks as a disease to be detected by various possible tests, we describe the ability of available tools to correctly identify “diseased cases” (positive predictive value) and the risk that they will miss such cases (negative predictive value). Insights from this analysis may provide guidance on how best to identify and shut down large-scale bot attacks on surveys.

## Methods

We conducted a secondary analysis of data from a survey of caregivers of children with chronic conditions in California. Results of the study have been previously reported [12-15].

### Population and Setting

We recruited caregivers of children with chronic conditions who were clients of 17 family resource centers (FRCs) across California. FRCs are nonprofit community agencies serving families of children with developmental delays and chronic medical conditions through education and advocacy. For example, they frequently provide classes and peer support to help caregivers of children with chronic conditions to secure social and educational services, and to connect with other families of children with similar conditions. Due to restricted budgets, they often have fewer organizational resources, including paid staff, and rely on volunteer caregivers to advise and provide services to families. They frequently communicate with clients through email listservs. Eligible participants were English- or Spanish-speaking adult caregivers residing in California whose child had a visit with a new pediatric subspecialist in the previous 12 months.

### Distribution of the Survey

The study team initially proposed sharing a list of unique, single-use survey links with FRCs to be distributed to their clients, but FRCs were unable to use these unique links with their listserv software and were restricted from sharing their listservs with outside organizations, like the study team, which could have sent unique links directly to FRC clients. As a result, we generated a unique generic link for each FRC. FRCs distributed an invitation to the survey from the study team and the survey link through their email listservs of clients and printed flyers with a QR code link to the survey to post in their offices for access by families coming in for services. Data was collected between August and October 2023.

### Fraud Detection Protocol

Based on the REAL (Reflect, Expect, Analyze, Label) framework for online survey fraud [16], we considered in advance how to prevent or identify fraudulent responses at each stage of the process, including pre–data collection (eg, recruitment through community-based partners), data collection (eg, integration of fraud detection questions in the survey design), and post–data collection (eg, a systematic review process). We also borrowed ideas from Anomaly Detection Theory, grounded in engineering sciences, which posits that detection of anomalies requires identifying data that do not follow established patterns [17].

We anticipated that the distribution of the survey through an anonymous link to large groups of people could make the survey vulnerable to fraudulent responses, and in particular, to bot attacks in which an individual writes code to automatically submit multiple survey responses that appear to originate from eligible respondents. Based on sparse literature and consultation with colleagues, the team utilized 10 different fraud detection tools when the survey was fielded, including proprietary tools that can be turned on within the Qualtrics survey platform, such as invisible ReCAPTCHA technology, and other flags, like the Relevant ID Duplicate, Fraud scores, and Ballot Box Stuffing flags. In addition, we embedded questions that required internally consistent logic (eg, birth year and age), monitored open-ended questions for nonsensical or repeating responses, reviewed longitude and latitude to ensure respondent location in California, considered records based on completion times (including shorter-than-expected response time and duplicate submission times), and verified IP addresses of respondents through an external server of suspicious addresses (see Multimedia Appendix 2). The survey also included a series of 13 screening questions designed to remove ineligible participants before reaching the body of the survey, which we believed would discourage fraudulent responses.

**Figure 1.**
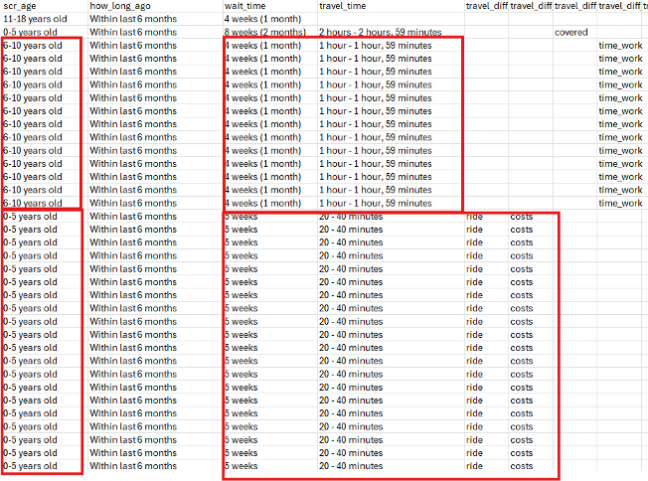
Examples of fraudulent response patterns (repetition of blocks across multiple records) in a cross-sectional survey of caregivers of children with new pediatric subspecialty referrals (August-October 2023).

The number of responses was monitored multiple times each day, and spikes in responses were immediately investigated. We completed a logbook entry for responses that triggered concern. Most indicators of fraudulent activity have caveats; for example, IP addresses triggering warnings from the fraudulent database could result from a previous infection of a legitimate device by malware that does not affect the survey (see Multimedia Appendix 2 for additional examples). As a result, no single flag was treated as a sole determinant of fraud. Classification of all records with flags was conducted through consensus between a senior researcher and a research associate. Fraudulent responses were often confirmed through patterns of responses, such as blocks of questions with the same responses (see Figure 1 for example). In the minority of cases in which the coding team was uncertain about a determination, the research associate followed up with the participant, requesting additional information that they could check against the survey to confirm that the response was valid.

### Data Analysis

We cleaned datasets from each FRC and reviewed records for final disposition (eg, valid, ineligible based on various criteria, fraudulent). We examined differences in demographic characteristics and health care utilization (outcomes in the primary study) between valid and fraudulent records in order to ascertain how our results might differ if fraudulent data were not identified and removed.

**Figure 2.**
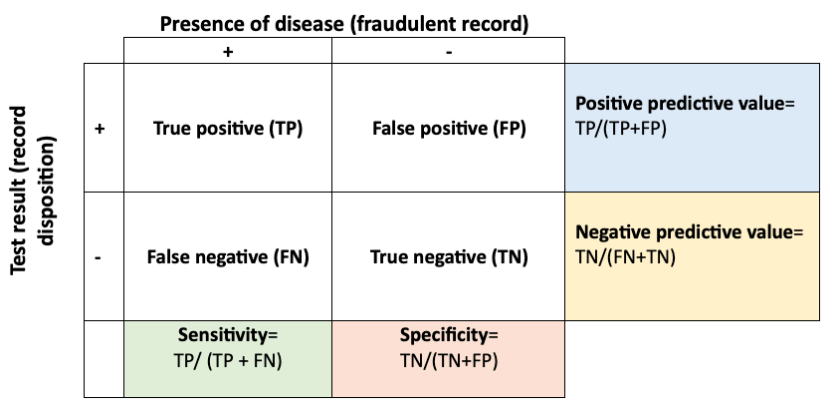
Epidemiological measures of diagnostic test accuracy.

To assess the validity of different fraud detection methods, we calculated the sensitivity, specificity, and positive and negative predictive value for each method (see Figure 2). Sensitivity is the ability of a test or method to correctly identify individuals with a disease, in this case, fraudulent records, while specificity is the ability of a test to correctly identify individuals without a disease, here, valid records [18]. Epidemiologists do not define universal thresholds of acceptable sensitivity and specificity, taking into consideration the danger of having false-positive or negative findings based on the severity and prevalence of the condition, for example. However, one rule of thumb is that the sum of the two values should be between 150% and 200%, meaning that it is at least more likely than chance alone to result in a correct identification [19]. We accordingly calculated the sum of sensitivity and specificity.

Positive predictive value is the proportion of individuals testing positive (given a fraudulent study disposition) who actually have the disease (are actually fraudulent). Negative predictive value is the proportion of individuals testing negative (given a valid disposition) who truly do not have the disease (are, in fact, valid records). Both positive and negative predictive values depend not only on the sensitivity and specificity of the tests, but also on the prevalence of the condition in the population (in this case, the prevalence of bot attacks in the pool of survey records).

### Ethical Considerations

The protocol for the original study was approved by the University of California, San Francisco (UCSF) Institutional Review Board (23‐39316). The board approved implied consent for the study, whereby participants clicking on the survey link first viewed an information page approved by the board and then opted into participation by clicking on a link to take part in the survey. Data was deidentified prior to analysis. Respondents received a US $30 electronic gift card for completing the survey, once the validity of the survey was confirmed by the study team (see Multimedia Appendix 3).

## Results

Of 2220 records, 646 (29%) were valid and complete (hereafter “valid”), 905 (41%) were classified as fraudulent by the protocol, 263 (12%) were valid but ineligible based on the eligibility screeners, 308 (14%) were incomplete surveys, and 98 (4%) were duplicate records. Duplicate records were distinguished from fraudulent records as those that made no effort to disguise their identity or device as identical; these appeared to result from respondents being unsure if their previous response had been received. Subsequent analyses were limited to valid and fraudulent records (n=1551).

There were a number of statistically significant differences in the reported demographics of valid versus fraudulent records (Table 1). When valid responses were compared with the overall responses (for valid + fraudulent records), valid responses were more likely to report having younger children (317/646, 49.1% aged 0‐5 y vs 536/1472, 36.4% for all responses); being Asian American and Pacific Islander (106/639, 16.4% vs 125/1368, 8.1%) or Latino/a (352/639, 55.1% vs 382/1368, 27.9%); living in a big city (218/635, 34.3% vs 697/1343, 51.9%); and having commercial health insurance (226/640, 35.3% vs 344/1353, 25.4%).

**Table 1.** Demographic characteristics of all, valid, and fraudulent respondents to a cross-sectional survey for caregivers of children with new pediatric subspecialty referrals in California (August-October 2023).

Characteristic	All (valid + fraudulent records), n/N (%)	Valid records, n/N (%)	Fraudulent records, n/N (%)	Difference between valid and fraudulent records	
				Pearson chi-square (*df*)	*P* value
Age (y)				104.12 (1)	<.001
0-5	536/1472 (36.4)	317/646 (49.1)	219/825 (26.5)		
6-10	527/1472 (35.8)	148/646 (22.9)	379/825 (45.9)		
11-20	408/1472 (27.7)	181/646 (28.0)	227/825 (27.7)		
Child’s gender^a^				1.41 (1)	.24
Boy	829/1364 (60.8)	398/640 (62.2)	431/724 (59.5)		
Girl	530/1364 (38.9)	237/640 (37)	293/724 (40.5)		
Another gender	5/1364 (0.4)	5/640 (0.8)	0/724 (0.0)		
Race/ethnicity^b^					
Black/African American	190/1368 (13.9)	38/639 (5.9)	152/728 (20.9)	63.4 (1)	<.001
American Indian/Alaskan Native	34/1368 (2.5)	31/639 (4.8)	3/728 (0.3)	27.7 (1)	<.001
Asian/Pacific Islander	125/1368 (8.1)	106/639 (16.4)	19/728 (2.1)	104.2 (1)	<.001
Hispanic/Latino	382/1368 (27.9)	352/639 (55.1)	30/728 (4.1)	439 (1)	<.001
White	799/1368 (58.5)	258/639 (40.4)	541/728 (74.3)	161.4 (1)	<.001
Other	20/1368 (1.5)	18/639 (2.8)	2/728 (0.3)	—^c^	—
Urbanicity				149 (2)	<.001
Big city	697/1343 (51.9)	218/635 (34.3)	479/708 (67.7)		
Small city or suburb	557/1343 (41.5)	358/635 (56.4)	199/708 (28.1)		
Rural area	89/1343 (6.6)	59/635 (9.3)	30/708 (4.2)		
Insurance^b^					
Medicaid	986/1353 (72.9)	488/640 (76.3)	494/713 (69.9)	7 (1)	.008
Commercial	344/1353 (25.4)	226/640 (35.3)	118/713 (16.6)	62.6 (1)	<.001

a Reflects caregiver-reported gender. Because of small cell sizes, “another gender” was excluded from testing of differences across groups.

b May report more than 1 response, so responses may sum to more than 100%. For multiple response comparisons, used Pearson *Χ*2 analysis to compare responses for each option across valid versus fraudulent responses.

c Not applicable.

With respect to health care utilization, which was the key outcome in the primary study, there were significant differences between valid versus fraudulent responses. Valid responses generally reported lower utilization than overall responses, such as having at least one hospitalization in the last year (172/639, 26.9% for valid vs 578/1352, 42.8% of overall responses; Table 2), at least one emergency department visit (324/638, 50.8% vs 799/1351, 59.1%), and at least 1 hour per week in medical visits (469/636, 73.7% vs 1139/1347, 84.6%). On the other hand, valid respondents were more likely to report 3-month waits or more for pediatric subspecialty care compared to the overall sample (156/638, 24.5% vs 271/1382, 19.6%).

**Table 2.** Outcomes for health care utilization of all, valid, and fraudulent respondents to a cross-sectional survey for caregivers of children with new pediatric subspecialty referrals in California (August-October 2023).

Outcome	All (valid + fraudulent) records, n/N (%)	Valid records, n/N (%)	Fraudulent records, n/N (%)	Difference between valid and fraudulent records	
				Pearson chi-square (*df*)	*P* value
Hospital visits in last year				124.1 (1)	<.001
0 times	774/1352 (57.3)	467/639 (73.1)	307/713 (43.1)		
1 or more times	578/1352 (42.8)	172/639 (26.9)	406/713 (56.9)		
ED^a^ visits in last year				34.9 (1)	<.001
0 times	552/1351 (40.9)	314/638 (49.2)	238/713 (33.4)		
1 or more times	799/1351 (59.1)	324/638 (50.8)	475/713 (66.6)		
Wait time for pediatric subspecialist				17.6 (1)	<.001
2 mo or less	1111/1382 (80.4)	482/638 (75.6)	629/744 (84.5)		
3 mo or more	271/1382 (19.6)	156/638 (24.5)	115/744 (15.5)		
Hours per week in medical visits				108 (1)	<.001
<1 h/wk	208/1347 (15.4)	167/636 (26.3)	41/711 (5.8)		
1 or more hours/week	1139/1347 (84.6)	469/636 (73.7)	670/711 (94.2)		

a ED: emergency department.

We examined the validity of different fraud detection methods using epidemiological measures of diagnostic tests (Table 3). Most methods had low sensitivity (poor ability to detect fraudulent records), including comparisons of paired items (eg, age and y of birth), short completion time, duplicate IP addresses, and review of open-ended responses for duplicate or nonsensical answers. Even ReCAPTCHA technology, which relies on information that bots can see but humans cannot, only detected about half of fraudulent responses. The most sensitive indicators appeared to be a fraudulent IP address detection service, where a low threshold setting (ie, one that identified not only IP addresses with confirmed fraudulent activity but also those with suspicious activity) yielded a sensitivity of 88%, and the proprietary Fraud ID feature on the survey software, with a sensitivity of 67%.

**Table 3.** Sensitivity, specificity, positive and negative predictive value of fraud detection methods at identifying fraudulent responses in a cross-sectional survey for caregivers of children with new pediatric subspecialty referrals (August-October 2023) (n=1551).

Method of detecting fraudulent responses	True positive, n	False positive, n	False negative, n	True negative, n	Sensitivity (%)	Specificity (%)	Sensitivity + specificity (%)	Positive predictive value (%)	Negative predictive value (%)
Nonsensical open-ended responses	0	0	905	646	0	100	100	—^a^	41.7
Repeated open-ended responses	0	0	905	646	0	100	100	—	41.7
Qualtrics Ballot Box stuffing	0	0	905	646	0	100	100	—	41.7
Duplicate IP addresses	82	45	823	601	9.1	93	102.1	64.6	42.2
Short completion time (<4 min)	135	1	770	645	14.9	99.8	114.7	99.3	45.6
Discrepancy between paired items (age and year of birth)	183	15	642	631	22.2	97.7	119.9	92.4	49.6
Fraudulent IP address database (lower threshold)^b^	758	337	106	309	87.7	47.8	135.5	69.2	74.5
Longitude/latitude	389	9	637	516	37.9	98.3	136.2	97.7	44.8
Fraudulent IP address database (higher threshold)^b^	422	8	442	638	48.8	98.8	147.6	98.1	59.1
Qualtrics ReCAPTCHA functionality	466	0	439	646	51.5	100	151.5	100	59.5
Qualtrics Fraud ID	609	27	296	619	67.3	95.8	163.1	95.8	67.7
Combination: Qualtrics ReCAPTCHA or Fraud ID	675	37	230	609	74.6	94.3	168.9	94.8	72.6

a Not available.

b The service used to look up fraudulent IP addresses had two thresholds: a lower threshold indicating that the IP address had been used in suspicious activity, and a higher threshold, indicating that it had been used in fraudulent activity.

Sensitivity is typically balanced against specificity, the ability of a diagnostic test to accurately predict which people do not have a disease (in this case, to identify valid records). Almost all individual indicators yielded relatively high specificity, suggesting that they correctly identified the valid records in the sample, even as most of these methods could not identify the fraudulent ones well (Table 3).

When we examined the sum of sensitivity and specificity and used the proportional threshold of 150% or greater as an indicator of the ability of a test to identify cases, only fraud detection tools embedded in the survey software (ReCAPTCHA and Fraud ID within the Qualtrics system) met the criteria of being more useful than chance alone in identifying fraudulent records. Notably, each of these methods used alone would have missed between 33% and 48% of fraudulent records, based on their modest sensitivity. Using the two measures together (ie, flagging if either was positive) would have resulted in a sensitivity of 75% and a specificity of 94%, with a combined total of 168.9%.

When we calculated predictive values for each method, we found that several had positive predictive values in excess of 90%, meaning that if they were activated (a positive test), it was a fairly good indicator that a record was indeed fraudulent, but negative predictive power less than 60%, indicating that the failure of a record to activate one of these indicators was not assurance that the record was valid. For example, activation of the survey software’s ReCAPTCHA indicators was strongly suggestive of a fraudulent record (positive predictive values of 100%), but its negative predictive value (59.5%) suggests that it would miss around 40% of fraudulent records.

## Discussion

Similar to past studies [1,3,5-10], in this large statewide survey, we observed a high rate of fraudulent records compared to valid ones (1.4 fraudulent records for every valid record) that would have significantly altered the characterization of the sample and study findings if undetected. Strategies recommended for survey fraud detection performed poorly as judged by epidemiological standards for detecting the “disease” of fraud. These included a number of tried-and-true methods, such as the use of paired items (age and year of birth) at opposite ends of the survey [2,5,8-10], which detected only 1 in 5 fraudulent records, or looking for duplicate IP addresses, which detected only 1 in 10 fraudulent records. Only 2 methods, both proprietary survey software technologies, performed better than chance alone in identifying fraudulent surveys, but even the combination of the 2 methods failed to detect about a quarter of fraudulent survey responses.

Fraudulent responses, if undetected, would have significantly altered the characterization of the sample as well as the study findings. For example, the responses from bot attacks indicated greater health care utilization and shorter wait times for pediatric subspecialty care compared to valid responses. Thus, failure to remove bot responses would have resulted in the incorrect conclusion that Californian families have greater access to pediatric subspecialty care than they actually do.

None of the 10 recommended fraud detection methods used in this study was effective as a stand-alone method capable of identifying fraudulent records, even those that other studies have reported to be highly effective. Moreover, our study represents a small number of bot attacks, and comparison to past literature suggests that bot attacks can have highly individualized signatures. For example, MacKinnon et al [20] were able to identify that over 31% of responses were fraudulent based on nonsensical open-ended questions, duplicate IP addresses, geocoding, and short completion time. In our study, these combined methods would have detected only 43% of fraudulent cases. Rather than use our results to suggest discarding or prioritizing particular measures, we echo the call of Dewitt et al [6] for the use of dynamic protocols, which use as many methods as possible to flag suspicious cases and human reviewers to manually review flagged records. The method is analogous to installing motion sensors, alarms at entry points, and safe meddling safeguards at a bank, but activating human guards to investigate if any of these detection mechanisms are activated.

Combating fraudulent survey responses poses a fundamental tension between data quality, inclusion, and resources. Manual review of responses and follow-up with suspect cases was highly labor-intensive in this study. Yet fraud detection protocols that require prior authentication of participants (eg, through requiring participants to upload documents [3]) or seek to expeditiously classify cases based on a priori algorithms risk exclusion of legitimate respondents. Populations with lower income, racial and ethnic minorities, and older adults are already underrepresented in internet research [21-23]. Adding additional barriers may pose not only an inconvenience but also a threat to the inclusion of historically marginalized voices [3]. In the short term, combating bot attacks will require greater allocation of resources. Developing strategies to improve bot detection, reviewing results in real time, and following up with participants when the authenticity of responses is in question are time-consuming and raise the cost of research. Ultimately, the field must invest in developing more cost-effective strategies for identifying and removing fraudulent records.

Advances in artificial intelligence (AI) will likely increase the frequency and sophistication of bot attacks, including mimicking human response biases, in an effort to evade detection. On the other hand, they may also contribute to the development of important bot detection tools that can co-evolve with bot attacks. Machine learning models may be well equipped over time to identify newly emerging patterns of fraudulent activity and to adapt as fraudulent tactics evolve. However, the emerging tool of AI does not make current research on patterns of fraudulent survey activity irrelevant, because development of technology for detecting bot attacks will initially rely on existing datasets, such as the one used for this study, using human classification of responses as valid or fraudulent. Anomaly Detection Theory advances a number of approaches to identify problem cases, such as a search for outliers [17]; however, when there are more fraudulent than valid responses to a survey, as was the case with our survey and has been with others, fraudulent cases may cease to be outliers. The need for accurate data for learning models may, therefore, lend even greater urgency to accurate identification within the datasets that are fed to the AI models.

Limitations of this study include a lack of a true gold standard for identifying fraudulent responses, which meant that we relied on manual review and consensus determination of case dispositions as reflective of the correct disposition of fraudulent or valid. On the other hand, we employed a high-effort strategy to distinguish valid from fraudulent responses, reviewing each response manually and following up with respondents when the validity of the response was in doubt, and we observed patterns unlikely to have occurred through true responses. Because fraud detection technologies such as Qualtrics ReCAPTCHA are proprietary, we cannot offer insight into the mechanisms of their ability to detect some fraudulent attacks or their failure to detect others. We collected data in August–October 2023, and bot-facilitating technology and methods may have evolved since that time. This study was conducted through web-based surveys using anonymous links shared through community-based organizations serving families of children with chronic conditions. Further study is needed to generalize findings to understand patterns of response as bot attacks become more sophisticated.

The incidence of fraudulent responses to web-based surveys using bots has been rapidly increasing in survey research and threatens the integrity of important research. Our study identified two methods that performed better than chance alone in identifying fraudulent cases; the combination of these two tools, both proprietary tools of the survey software, identified 75% of fraudulent cases. Literature suggests that bot attacks may have diverse phenotypes, and our findings support calls for “dynamic protocols” that capture as many potential markers of fraud as possible for review and follow-up. To avoid the need to expend intensive human resources in future research, it is imperative that researchers begin to employ more sophisticated technology, investing in AI-enabled bot detection approaches that can evolve along with fraudulent attacks. As such, interdisciplinary collaborations with computer scientists and tech entrepreneurs who currently solve security problems could be fruitful to elevate the voices of real people within policy and programmatic discussions.

## Supplementary material

10.2196/85161 Multimedia Appendix 1 Literature review, including methods tested, and proportion of fraudulent responses.

10.2196/85161 Multimedia Appendix 2 Approaches to detecting fraudulent responses.

10.2196/85161 Multimedia Appendix 3 Sample language in institutional review board/consent form.

## Abbreviations

AI: artificial intelligence
FRC: family resource center
REAL: Reflect, Expect, Analyze, Label
