# Supplementary material for: Using Epidemiological Test Diagnostics to Select Fraud Detection Methods: Secondary Analysis of Quantitative Cross-Sectional Survey Data
Source: J Med Internet Res. 2026 Mar 5;28:e85161. doi: 10.2196/85161 (PMC12978920; doi:10.2196/85161)
Supplement: Multimedia Appendix 1 [file jmir-v28-e85161-s001.docx]

**Appendix 1: Literature review, including methods tested, % fraudulent responses**

| **Author** | **Year** | **Strategies tested** | **Fraudulent responses** | **Total responses** | **% fraudulent responses** |
| --- | --- | --- | --- | --- | --- |
| Campbell [3] | 2022 | ReCAPTCHA, geolocation, documentation of age and serostatus through uploaded docs | 413 | 739 | 55.9% |
| Pratt-Chapman [4] | 2021 | CAPTCHA, hidden question, instructions to type specific word, asked how heard about survey, time stamps, pairs of items, used 2-flag method to identify suspicious records. Appeared to use a curing process as well. | 1408 | 1977 | 71.2% |
| Choi [5] | 2017 | Incentives changed from gift card to raffle, blocking multiple entries from single IP addresses, masking eligibility, paired items for consistency, statements about data surveillance. | 146 | 342 | 42.7% |
| Dewitt [6] | 2018 | Baker and Downes-LeGuin standards (8 indicators: Short completion, selection of all items in multiple response, selection of bogus or low probability answers, internal inconsistencies, straight lining, high levels of non-response failure of verification items, gibberish in open ended; 3 strikes rule) -- modified, but similarly no one indicator was sufficient to call a survey invalid. | 289 | 478 | 60.5% |
| Griffin [7] | 2021 | Use of ReCAPTCHA, bot detection; change from incentive per completion to raffled gift cards; breaking up screening questions from survey; 5 steps cleaning protocol: a) remove incomplete responses (<60%); b) reCAPTCHA; c) remove outliers for response time; d) duplicate qualitative responses; e) internal inconsistencies; f) duplicate email or IP addresses; g) suspicious emails (>4 #s) | 773 | 1251 | 61.8% |
| Hardesty [18] | 2024 | Short completion times, surveys taken without first clicking social media ad, duplicate email address, high number of “don’t know” responses, duplicate photos, use of non-English alphabet, ad-location mismatches, unusually high agreement across responses to multiple surveys, inconsistent responses to matched items | 1,261 | 1624 | 77.6% |
| MacKinnon [19] | 2025 | Inclusion of fake medical conditions in response options, nonsensical or hateful email addresses, multiple IP addresses, location outside of U.S. or Canada, non-matching survey items, short completion time, use of phone calls with people whose survey entries were categorized as suspicious | 420 | 1,377 | 30.5% |
| Mournet [8] | 2023 | Conducted 4 rounds of data collection; removed mention of compensation in last round and got fewer bot attacks. Used Orabi et al method: a) reporting where heard about study (match to actual methods); b) text responses do not equal info from consent; c) intelligible open ended responses; d) matched items compared; e) look for similarities in open ended responses across respondents; f) enough open ended responses to feel confident in integrity of data. Response was considered fraudulent if failed any of these tests. Also used ReCAPTCHA and items where they told people which answer to choose ("attention checks"). They also used bit.ly to create a URL without "Qualtrics" in order to "disguise" their survey from bots. Table 1 tabulates reasons that fraudulent surveys were flagged. They also used follow up emails when in doubt about authenticity. They noted that they got follow up emails about compensation that appeared to be bot generated. | 340 | 359 | 94.7% |
| Myers [2] | 2022 | Survey aimed at Sexual and Gender Diverse audience in New Mexico. Used Every Door Direct Mail, Google and Facebook ads, organizational outreach, flyers at clinical and community settings. Identified 4 criteria for fraudulent surveys (duplicate email, matches in qual responses of more than 3 words, zip codes not in U.S., heterosexual and cisgender; and 17 criteria for suspicious surveys (e.g., zip codes out of area, height or weight extremes, nonsensical qualitative responses, incongruence SAAB-SOGI-organs - More in Table 1). Records with 1 fraudulent criterion or 3 or more suspicious criteria were removed. They did advertise an incentive. | 406 | 3120 | 13.0% |
| Pekarsky [9] | 2022 | Defined fraudulent responses as records with made up names and email addresses or location outside of Calgary (based on IP addresses). Also looked at internal consistency (gestation age no matching due date), nonsensical text, phone numbers or IP addresses outside Calgary. They also kicked people out if their name and email didn't match. Ultimately removed mention of incentive from announcement. Later added logic checks, monitoring for fake email addresses, duplicate IP addresses, adding screening questions with contact info and demographic info. | 1220 | 1572 | 77.6% |
| Pozzar [10] | 2020 | Eligibility included CAPTCHA, asking how heard of study, monitoring time for survey completion, hidden items visible only to bots, pairs of items (e.g., timestamp time zone and self-reported location), duplicate or unusual responses to open ended questions, inconsistent responses to verifiable items. Time of completion (i.e., group of responses in early morning) was clue that there was a bot attack, but it was not one of the criteria for fraud because it was not certain that completion between 1-5 AM meant a given response was fraudulent. | 256 | 271 | 94.5% |
| Ruby [20] | 2025 | Same timestamp and similar responses, fraudulent email address provided, suspicious or short timestamp | 2,440 | 2,494 | 97.8% |
| Zuniga [1] | 2023 | Looked at duplicate IP addresses, inconsistent responses, suspicious or nonsensical responses. | 162 | 167 | 97.0% |
|  |  | **Overall** | **5413** | **10276** | **60.5%** |
