# Supplementary material for: Using Epidemiological Test Diagnostics to Select Fraud Detection Methods: Secondary Analysis of Quantitative Cross-Sectional Survey Data
Source: J Med Internet Res. 2026 Mar 5;28:e85161. doi: 10.2196/85161 (PMC12978920; doi:10.2196/85161)
Supplement: Multimedia Appendix 2 [file jmir-v28-e85161-s002.docx]

**Appendix 2: Approaches to detecting fraudulent responses**

| **Approach** | **Description** | **Caveats** |
| --- | --- | --- |
| Duplicate IP addresses | Entries from the same IP address (suggesting use of the same device, so likely more than 1 entry for individual) | It is not impossible that multiple families could use a single device (e.g., a library computer) |
| Fraudulent IP address database* | Ran IP addresses of respondents through database of IP addresses associated with known fraudulent activity | Legitimate personal devices can be used as part of botnets, unbeknownst to their owners |
| Nonsensical open ended responses | Response to open ended questions that are not related to the question | Need to ensure not to conflate hurried text or phrases used by legitimate respondents with primary language other than English |
| Repeated open ended responses | Duplicated open ended responses across multiple surveys | Requires complexity of response to ensure (i.e., the responses “nothing” or “N/A” are commonly repeated but not an indication of fraud) |
| Discrepancy between paired items (age and year of birth) | Placement of 2 questions whose responses should align at different parts of the survey (e.g., age and year of birth) | If the 2 items are close, could be a result of legitimate miscalculation |
| Bot detection technology (e.g., Qualtrics reCAPTCHA feature) | Proprietary technology to identify bot activity | Technology is proprietary, so it is not clear what it relies on |
| Prevention of duplicate entries (survey software functionality) | Survey software blocks or flags entries from the same IP address | It is not impossible that multiple families could use a single device (e.g., a library computer) |
| Short completion time (Less than 4 minutes) | Total completion time is less than 4 minutes | Need to select threshold at lowest end of range of realistic time |
| Longitude/latitude of completion outside of survey region | Survey software provides the longitude and latitude where the survey was completed; compared with outer boundaries of geographic target zone | Legitimate respondents can travel |

* The fraudulent email address detection service used two different thresholds (possibly and likely fraudulent), which are set by the external site and are proprietary (not public). In analyses, we examined these two thresholds separately to determine the positive and negative predictive value of each threshold.
