# Supplementary material for: Using Epidemiological Test Diagnostics to Select Fraud Detection Methods: Secondary Analysis of Quantitative Cross-Sectional Survey Data
Source: J Med Internet Res. 2026 Mar 5;28:e85161. doi: 10.2196/85161 (PMC12978920; doi:10.2196/85161)
Supplement: Multimedia Appendix 3 [file jmir-v28-e85161-s003.docx]

**Appendix 3: Sample language in IRB/consent form**

**In IRB application**

Within procedures: Because of a concern about bots completing surveys that we have been alerted to in other settings, we will take several measures to discourage or identify and remove responses that appear to come from bots.

1. We will distribute unique survey links when possible, such that a single link cannot be used to take the survey multiple times.
2. When an anonymous link to the survey must be used, we will enable a Qualtrics feature to "prevent ballot box stuffing" that disables multiple survey entries from 1 IP address
3. We will avoid distribution on social media or other means that are more vulnerable to bots
4. We will use Captcha and bot detection features on Qualtrics to discourage bot responses
5. We will run IP addresses through an IP address fraud checker (scamalytics.com)
6. We will look for several other signals of bot responses, including:
7. clusters of responses from the same IP address or with identical information
8. responses to open ended questions that are unrelated to the subject matter (e.g., a colleague identified bot targeting of a survey when respondents to a survey about legal needs garnered responses about wait staff service quality)
9. contradictions in duplicated questions

"Fraudulent" responses are defined as:

- Those linked to an IP address that is known to be connected to fraudulent activity (e.g., those identified by Scamalytics)
- Those with non-sensical open ended responses that appear to be bot generated (e.g., restaurant critiques on a survey about health services)
- Clusters of at least 3 responses from single IP addresses

"Suspicious" responses are defined as: Contradictions between questions.

In the event that we determine that a response is flagged as "fraudulent," we will remove them from the data and seek to shut down any links that appear vulnerable. In the event that responses are flagged as "suspicious," we will seek to contact the individuals, alert them that we flagged their case as a potential false response and provide them with a phone number to contact if they were a legitimate respondent.

**In consent form:** All entries will be reviewed for evidence of fraudulence, which means enrolling in the study dishonestly and pretending to meet study criteria when not actually eligible. If fraud is detected, you will be removed from the study without remuneration which is receiving a gift card.
